# The evolution of costly acquired immune memory

**DOI:** 10.1002/ece3.611

**Published:** 2013-06-06

**Authors:** Alex Best, Andy Hoyle

**Affiliations:** 1 Mathematics and Statistics, University of SheffieldSheffield, S3 7RH, U.K; 2 Biosciences, University of Exeter, Cornwall CampusPenryn, TR10 9EZ; 3 Computing Science and Mathematics, University of StirlingStirling, FK9 4LA

**Keywords:** Acquired immunity, host-parasite, immune memory, SIR

## Abstract

A key feature of the vertebrate adaptive immune system is acquired immune memory, whereby hosts launch a faster and heightened response when challenged by previously encountered pathogens, preventing full infection. Here, we use a mathematical model to explore the role of ecological and epidemiological processes in shaping selection for costly acquired immune memory. Applying the framework of adaptive dynamics to the classic SIR (Susceptible-Infected-Recovered) epidemiological model, we focus on the conditions that may lead hosts to evolve high levels of immunity. Linking our work to previous theory, we show how investment in immune memory may be greatest at long or intermediate host lifespans depending on whether immunity is long lasting. High initial costs to gain immunity are also found to be essential for a highly effective immune memory. We also find that high disease infectivity and sterility, but intermediate virulence and immune period, increase selection for immunity. Diversity in host populations through evolutionary branching is found to be possible but only for a limited range of parameter space. Our model suggests that specific ecological and epidemiological conditions have to be met for acquired immune memory to evolve.

## Introduction

An adaptive immune system appears to exist in almost all vertebrates (Cooper and Alder 2006). A key feature of this defense mechanism is acquired immune memory, whereby hosts can protect themselves from subsequent infections from the same pathogen. This mechanism is incorporated in to the classic Susceptible-Infected-Recovered (SIR) epidemiological model through the assumption that upon recovery from infection, hosts gain long-lasting immunity to disease such that they cannot be re-infected (Kermack and McKendrick 1927; Anderson and May 1979). This adaptive immune response is perhaps the most advanced defense mechanism possessed by vertebrate hosts to natural parasites and pathogens. However, the factors that may impact on the evolution of such an immune memory from a theoretical perspective are still not fully understood.

Clearly, selection for any form of defense to parasitism is governed by the ecological and epidemiological environment of the host. A common prediction is that investment in defense should be monotonic with host lifespan (Medzhitov and Janeway 1997; Rinkevich 1999), but in a key theoretical study Miller et al. (2007) showed that this is not always the case. In particular, they found that if hosts could evolve the length of the immune period then investment was indeed greatest for long-lived hosts, but if permanent acquired immunity was evolved then investment was instead greatest at intermediate lifespans (Miller et al. 2007; see also van Boven and Weissing, 2004). Understanding the differing ecological feedbacks between these two models is therefore crucial to understanding the evolution of immunity in different host populations. Further to this work, van Baalen (1998) found that an effective clearance mechanism, crucial to the development of immune memory, was most likely against parasites with intermediate virulence, while Boots and Bowers (1999) showed that coexistence of types with high and low clearance levels was possible. Boots and Bowers (2004) further investigated the evolution of immune period, and found very similar results – long-lasting immunity was most likely against parasites with intermediate virulence and coexistence of types with short- and long-lasting immunity was possible. Recent theoretical work has also studied the evolutionary ecology of the maternal transfer of immunity (Garnier et al. 2012) and of immune priming in invertebrates (Best et al. 2013), yet there is still a clear need for a thorough investigation of the evolution of immune memory in vertebrate hosts.

The vertebrate adaptive immune system involves a complex set of genetic and molecular processes (Bonilla and Oettgen 2010). T and B lymphocytes, the main effector cells of the adaptive immune system, are activated to fight infection and produce long-lasting memory cells that are able to recognize specific antigenic configurations of previous pathogens. This immune memory then allows a faster immune response on subsequent challenges and the effective prevention of future infections. There is currently much interest in how the adaptive immune response first evolved in vertebrates from an immunological perspective (Cooper and Alder 2006; Litman et al. 2010; Hirano et al. 2011). The principle genes of the adaptive immune system have been identified in every jawed vertebrate that has been tested (Cooper and Alder 2006), suggesting that, while it has continued to be fine-tuned over subsequent evolutionary time, the adaptive immune system was probably first formed in the earliest vertebrates (Cooper and Alder 2006). Moreover, the differing structures of the adaptive immune system in jawed and jawless vertebrates suggests that these two groups have experienced different selection pressures for the development of the adaptive immune system (Cooper and Alder 2006; Litman et al. 2010). Furthermore, there is increasing evidence that both invertebrates (Little and Kraaijeveld 2004; Schmid-Hempel 2005) and plants (Spoel and Dong 2012) also have some form of specific, long-lasting immunity to infections. Given the seemingly universal prevalence of an adaptive immune system in vertebrate populations, there is clearly now little genetic variation in immune memory. However, there are still important insights to be gained for our understanding of how the ecological and epidemiological environment of hosts and parasites may have initially shaped selection for immune memory, of the apparent discrepancy in investment in immunity with host lifespan (Miller et al. 2007) and of the fundamental differences between populations with (SIR-type models) and without (Susceptible-Infected-Susceptible [SIS]-type models) immune memory.

Our focus here is to investigate the ecological and epidemiological conditions that would promote the evolution of an acquired immune memory in host populations. In developing such a theoretical model, there are a number of key assumptions we must decide upon. For example, is immunity perfect or imperfect (i.e., can immune individuals still become infected but at a reduced rate)? Furthermore, is immunity permanent or can it wane over time? There is also a subtle distinction in how we interpret the transition to immunity. We shall assume that some proportion of recovering hosts become immune, but is it predetermined that some hosts have a fully functioning immune system and others none, or do all hosts have an equal probability of becoming immune? This distinction is particularly important from an evolutionary perspective because it impacts on how the life-history costs of investing in immunity are incurred. If only certain hosts will ever become immune only those hosts should pay the costs, whereas if all hosts have the potential to become immune all hosts will pay a cost. Each of these assumptions is likely to play some role in determining the outcome of evolution. Here, as a first step, and to allow comparison with the work of Miller et al. (2007), we shall assume that immunity is perfect but that it can wane (while Miller et al. 2007; Model III) assumed immunity was perfect and permanent). We shall also assume that all hosts have the potential to become immune. As such, increased investment in immunity corresponds to a “stronger” immune system and a greater probability of becoming immune upon recovery. We shall also assume that there is a single host and single pathogen population, allowing us to focus more on the role of epidemiological feedbacks to evolution, but implicitly removing the role of specificity, a further key feature of the adaptive immune system (see Discussion).

## Modelling

Given the assumptions outlined in the Introduction, we model the population dynamics of Susceptible, Infected and Recovered (immune) hosts with the following set of ordinary differential equations (c.f. Miller et al. 2007),


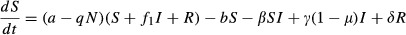
(1)


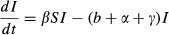
(2)


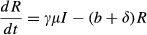
(3)

**Figure 1:**
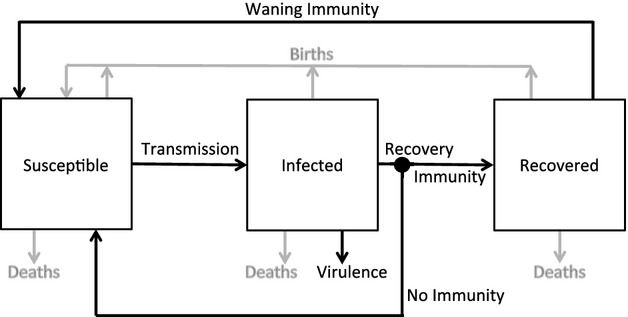
A schematic of our epidemiological model. The demographic processes of births and deaths are shown with gray lines, and the epidemiological processes of transmission, recovery, virulence, and waning immunity with black lines. The key “event” in our model is marked by the dot, where recovering hosts will either gain immunity and move to the recovered class, or will gain no immunity and return to the susceptible class.

The ecological and epidemiological processes of the model are also presented graphically in Figure 1 with a schematic of the model. All hosts are born susceptible at rate *a* (which is reduced through density dependence by *q*) but infected hosts may have reduced fecundity by a factor *f*_1_. We shall assume that immune hosts reproduce at the same rate as susceptible hosts throughout this article. All hosts die at natural mortality rate *b*. Transmission of disease is a mass-action process between susceptible and infected hosts with coefficient *β*. Infected hosts suffer increased mortality (defined as virulence) *α* and may recover from infection at rate *γ*. Upon recovery, a proportion, *μ* of hosts become immune, while the remainder do not gain immunity and return to susceptibility. As stated in the Introduction, we will assume that immunity is perfect, such that immune hosts have no risk of infection. However, we do assume that immunity can be lost at rate *γ*. Analysis of these population dynamics shows that this model yields straightforward SIR-type behavior, with a forward bifurcation at 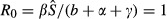 (where 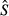 is the disease-free equilibrium) with one unique endemic equilibrium for *R*_0_ > 1. We note that the key differences between our model and model III of Miller et al. (2007) are that we allow immunity to wane and that we allow infected hosts to be sterilized.

We shall consider the evolution of becoming immune by allowing the parameter *μ* to evolve in the population. Following the standard methods of host evolutionary studies, we will assume that the cost of investment in defense, specifically immunity, is to the birth rate of the host, *a*. Note that all hosts pay this cost, not just those that are in the *R* class. Our focus is on how immunity may initially evolve in a host population and how strong the resulting immune memory will be, or, in other words, how populations may move from an SIS framework (i.e., recovery but not immunity) toward an SIR framework. It is likely that any initial investment in immunity would be particularly costly to create the necessary genetic and physiological structures for immune memory. We shall therefore assume that the trade-off curve is initially steep, with a small increase in immunity causing a large drop in reproduction, with costs decelerating at higher levels of immunity. Noting that *μ* must fall within the interval [0,1], we can express this trade-off as,


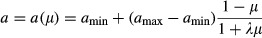
(4)

where the parameter *λ* controls the curvature of the trade-off. This produces a smooth, decreasing curve between extreme ends of the trade-off (*μ*_max_) and (*μ*_min_, *a*_max_). We take *λ* > 0 or the shape we require, which we refer to as “decelerating” in terms of costs, and consider its “strength” to mean how strongly curved the trade-off is (i.e., how steep the initial decline in reproduction).

As we are concerned with how the epidemiological processes feedback to the selection pressure for immunity, we will use the evolutionary framework of adaptive dynamics (Geritz et al. 1998). As such, we assume that a resident strain at equilibrium, with traits (*μ*_*r*_, *a*_*r*_), is invaded by a rare mutant whose trait values differ slightly from the resident (*μ*_*m*_, *a*_*m*_). The success of the mutant is governed by its invasion fitness, defined as its growth rate when rare. We use the Next Generation Matrix approach (Hurford et al. 2010) to calculate the fitness. We decompose the Jacobian of the mutant dynamics in to the form *J = F−V* where,


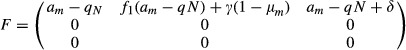
(5)

is a matrix containing terms relating to the creation of susceptible hosts from each host class, and,


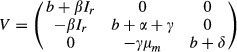
(6)

is a matrix containing terms involving the transition of individuals between classes and the death of hosts. Note that we have counted recovering infecteds, who are not immune and hosts who have lost their immunity as creation of susceptible host terms in the matrix *F* for analytical ease, but these matrices still conform to the conditions for using the next generation matrix (Hurford et al. 2010). The fitness of a mutant host is then *ρ*(*FV*^−1^)−1 where *ρ* denotes the spectral radius of the matrix. This produces a fitness as shown below,


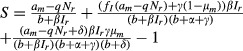
(7)

This fitness decomposes in to the (relative) contribution to the susceptible pool from each host compartment (*S*, *I* and *R*) weighted by the probability of hosts entering each compartment and the time spent in each compartment.

## Results

### When will the host invest in any immunity?

We first consider when a host population with no immunity will begin to invest in immune memory. As such we assume that initially *μ* = 0 (the system is SIS). The selection gradient at this point reduces as shown below,


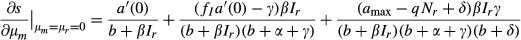
(8)

Note that *R* = 0 ⇒ *N* = *S* + 1. This clearly comprises two negative terms, associated with the marginal loss of reproduction as a cost (as well as the loss of hosts recovering to susceptibility) *a*^′^(*μ*) < 0, and a positive term associated with the marginal gain of increased reproduction from immune hosts. For hosts to evolve an immune memory, it is necessary that the above expression yields 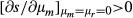 (i.e., there is a positive selection gradient at *μ* = 0. Noting that, *a*^′^(0) = −(*a*_max_ − *a*_min_)(1 + *λ*) and that (*b* + *α* + *γ*)/*β* = *S*_*r*_ the equilibrium susceptible density, this expression can be arranged to give the following:


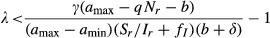
(9)

**Figure 2:**
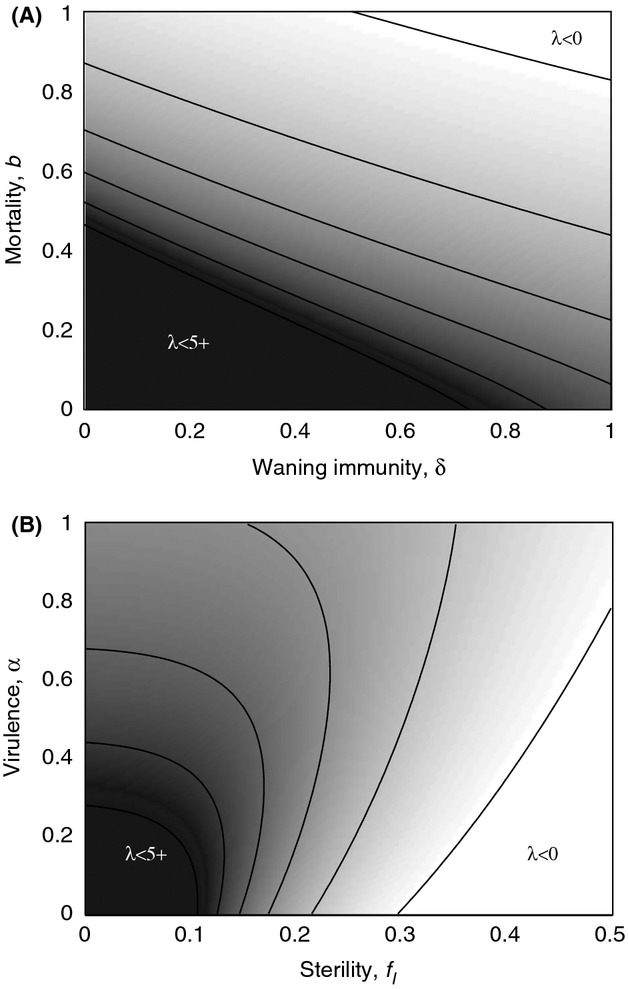
The range of trade-off curvatures for which equation (9) is satisfied, resulting in initial investment in immune memory. (A) Varying death rate, *b,* and waning immunity, *δ*, (B) varying virulence, *α*, and sterility, *f*_1_. Shading varies from white for *λ* < 0 to black for *λ* < 5 or more, with contours shown for greater clarity. Default values: *b* = 0.5, *α* = 0.5, *γ* = 0.5, *q* = 0.1, *β* = 2, *δ* = 0.1, *f*_1_ = 0.1, *a*_max_ = 5, *a*_min_ = 1.

The bigger the right-hand-side of equation (9), the greater range of trade-offs there are where the host will evolve an immune system; that is, the range incorporates “stronger” decelerating trade-offs, with steeper initial declines in reproduction. There will always be an upper limit on *λ* for immunity to evolve, as for initially very steep trade-offs the cost of gaining immunity (going from *μ* = 0 to *μ* > 0) will be too high. It is clear that most of the model parameters therefore influence the potential for immunity to evolve, both directly and through the term *S*_*r*_/*I*_*r*_. The shaded contour-plots in Figure 2 further explore this result, showing how the range of trade-offs for which immunity is evolved (i.e., for which equation (9) is satisfied) varies, from no decelerating trade-offs (*λ* < 0; white) to strongly decelerating trade-offs *λ* < 5 + ; black). In particular it can be seen that investment occurs for stronger trade-offs where hosts are long-lived (2A; small *b*) and immunity is long lasting (2A; small *δ*). This is to be expected as hosts with high death rates are unlikely to survive long enough to benefit from immunity, while if immunity is lost too rapidly its benefit is extremely limited. We also see that investment in immunity requires the parasite to be highly sterilizing (2B; small *f*_*I*_), but, generally, avirulent (2B; small *α*). High sterility causes a large fitness loss to hosts, increasing the selection for immunity. However, if virulence (that is, parasite-induced mortality) is too large, hosts are unlikely to recover from disease and therefore are unlikely to ever become immune.

### How much immunity will the host evolve? Epidemiological and ecological parameters

Having considered when immunity is likely to evolve at all, our focus turns to how much immunity the host will evolve. In other words here, how likely will it be that a recovering host becomes immune? A singular strategy of the system, a (potentially temporary) “stopping point” of evolution, occurs when the selection gradient is zero and there is no longer any directional selection (recall that *μ* ∈ [0, 1]. This can be expressed to give a condition in terms of the trade-off,


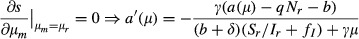
(10)

The stability of this singular point depends on two second-order terms. The strategy is evolutionarily stable provided 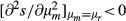, meaning that it is locally uninvadible. The strategy is convergence stable provided 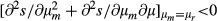, meaning that it is locally attracting.

**Figure 3:**
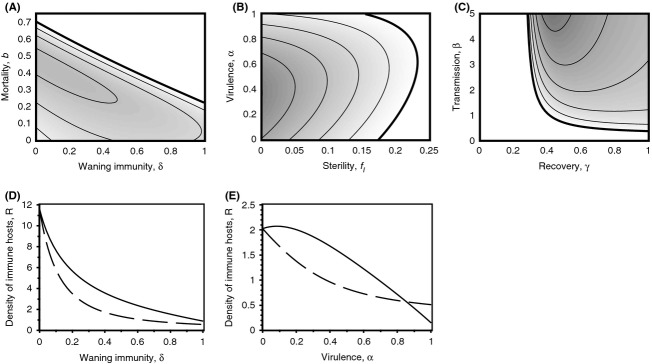
The continuously stable strategies (CSSs) level of investment in immunity. White regions indicate no immunity (*μ* = 0) with darker shadings indicating a greater level of immunity. For clarity, we plot contours for every 0.1 increment in investment (the bold line gives the *μ* = 0 contour). (A) Varying death rate, *b*, and waning immunity, (*δ*) (B) varying virulence, *α*, and sterility, *f*_1_ (C) varying recovery, *γ*, and transmission, *β*. We also show plots for how the density of immune hosts at the CSS (solid line) and a fixed level of *μ* (dashed line) for varying (D) waning immunity, *δ*, and (E) virulence, *α*. Default parameter values are as of figure 1 with *λ* = 2. In (D) *b* = 0.1.

We are initially focussing on the stable level of immunity hosts should invest in, and we therefore look for CSSs (continuously stable strategies) – singular strategies that are both evolutionarily stable and convergence stable and therefore long-term attractors of evolution (Eshel 1983; Christiansen 1991; Geritz et al. 1998). We therefore choose a gently decelerating trade-off curvature, *λ* = 2, that guarantees a CSS for the majority of parameter values tested. We shall consider the importance of the trade-off shape further in the next section. We again present our results using shaded contour-plots in Figure 3, this time showing the level of immunity invested in. White regions indicate no immunity (*μ* = 0) with darker shadings indicating a greater level of immunity.

Investment is greatest (darker colors) at intermediate or high host lifespans depending on the length of immunity (vertical axis, 3A). Short-lived hosts with high death rates are never likely to live long enough to benefit significantly from immunity so investment is always minimal. However, very long-lived hosts may only need to invest a small amount to ensure that they shall become immune at some point in their lives. This effect is amplified as the immune period 1/(*b* + *δ*) increases, as a low level of investment by long-lived hosts ensures they will be immune for a significant proportion of their life. We thus confirm the result from Miller et al. (2007) here, that investment is greatest at intermediate lifespans when immunity is permanent, as it is more important for these hosts to become immune earlier in their life. For intermediate- and short-lived hosts, investment is at its highest at low rates of waning immunity (horizontal, 3A) as would be expected. Interestingly, however, in long-lived hosts investment is in fact greatest at intermediate rates of waning immunity. We explore this result further in Figure 3D. The dashed line shows the purely epidemiological effects of increasing *δ* on the density of immune hosts where *μ* does not evolve (we fix *μ* be the CSS value at *δ* = 0), while the solid line shows the effect when *μ* does evolve to its CSS. Increasing waning immunity naturally acts to reduce the density of immune hosts. However, by increasing investment hosts are able to keep the density of immune hosts higher, leading to a greater contribution to fitness.

We see clearly that highly sterilizing parasites are needed for hosts to invest in immunity (horizontal, 3B), as otherwise the loss to fitness during infection is not significant enough to justify the investment. However, immunity is greatest at intermediate virulence rates (vertical, 3B). Again, we explore this in terms of the impact on the density of immune hosts where *μ* does not evolve (dashed line *μ* is fixed at the CSS value at *α* = 0) and where it does (solid line) in plot 3E. This shows that increasing virulence naturally acts to decrease the density of immune hosts. Again, though, by increasing investment in immunity this density can be kept at a higher level. However, for greater rates of virulence, hosts are killed quickly by disease and they are unlikely to recover and experience the benefits of immunity, so investment is dropped. Investment in immunity is clearly greatest against fast-transmitting parasites (vertical, 3C) as here disease prevalence will be highest. Investment is highest at intermediate rates of clearance (horizontal, 3C). If recovery is low hosts are unlikely to become immune and so investment is minimized, but if recovery is high hosts are unlikely to stay infected for long and, similar to that of virulence, the loss to fitness through sterility is not as great.

Overall, we see that there are large areas of parameter space where there is zero investment, and in fact we rarely see investment of *μ* > 0.5 for the gently decelerating trade-off shape studied. In general, evolution of a significant level of acquired immunity requires (i) intermediate or long host lifespans; (ii) high infectivity; (iii) intermediate virulence, and; (iv) high sterility.

### How much immunity will the host evolve? The trade-off

**Figure 4:**
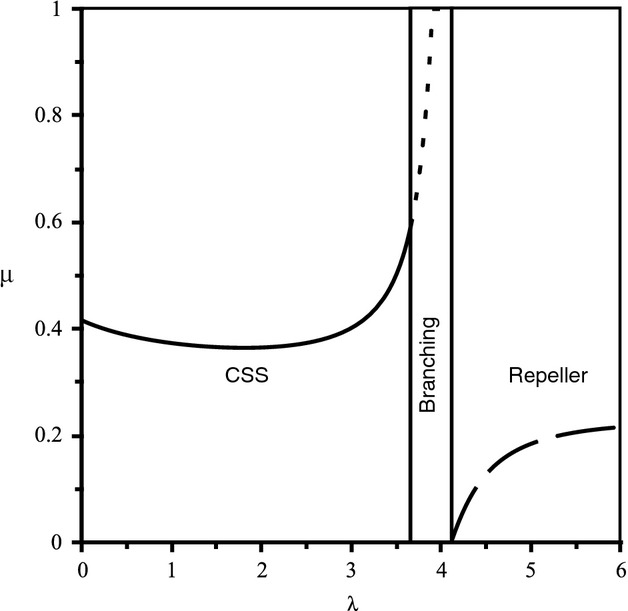
Location of the singular point and its stability for varying trade-off coefficient *λ*. The solid line denotes a continuously stable strategies (CSSs) (long-term attractor), the dotted line a branching point and the dashed line a repeller. Default parameter values as of Figure 1, but with *δ* = 0.05 and *β* = 2.5.

The strength of the trade-off also has a significant effect, not only on the level of investment but on the nature of the evolutionary outcome. Figure 4 shows, as one example, the effect of increasing the trade-off curvature on evolution. As the trade-off moves from linear (*λ* = 0) to gently decelerating, the CSS dips slightly before increasing at intermediate values of *λ*. The singular point then switches from being a CSS to a branching point. As the strength of the trade-off is increased further, the branching point moves beyond the maximum of *μ* = 1 and in this small region the population will remain monomorphic at maximum investment. There is then a discontinuity, and for very strongly decelerating trade-offs, the new singular point is a repeller, whereby the population will remain monomorphic but will move to minimum or maximum investment depending on the initial strategy.

**Figure 5:**
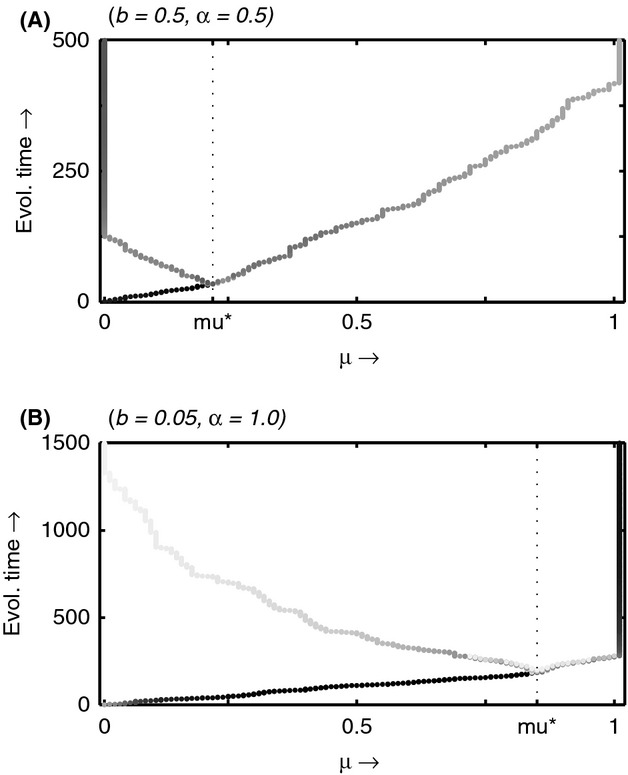
Numerical simulation of the evolution of immunity. In each case, we start at *μ* = 0 (representing no immunity in the population), it evolves toward a branching point and the population splits into two. The shading represents the density of the population (darker = higher). In (A) the resistant branch has a lower density than the nonresistant branch, whereas in (B) the resistant branch has a higher density. Default parameter values as of Figure 1, but with *δ* = 0.05. In (B) *α* = 1, *b* = 0.05.

Evolutionary branching provides one way in which full immunity can be achieved (in part of the population, at least). Here, hosts may approach a singular point that is convergence stable but evolutionarily unstable, causing hosts to undergo disruptive selection and branch in to two distinct strains. Subsequently the two strategies diverge and, in this scenario, evolve to extreme points, one with no immunity (*μ* = 0) and one with full immunity (*μ* = 1). An obvious question therefore is how these two strains compete against each other. Figure 5 shows simulation output for scenarios where evolutionary branching occurs. In the first simulation (5A), *μ* evolves to an intermediate (but low) value before branching – the subsequent strains diverge to achieve a fully immune sub-population (*μ* = 1) and a fully susceptible sub-population (*μ* = 0). The immune population exists at a lower density due to the high cost in birth rate they incur (as evidenced by the darker shading for that strain). However, in the second simulation (5B), where the parasite is more virulent and the hosts have a longer lifespan, branching takes place at a much higher value of *μ*; here it is the immune branch that exists at the higher density as hosts gain a much higher benefit from immunity, even with the associated costs to reproduction. Here (5B), hosts who develop immunity dominate the population whereas those with no immunity only exist at very low levels.

**Figure 6:**
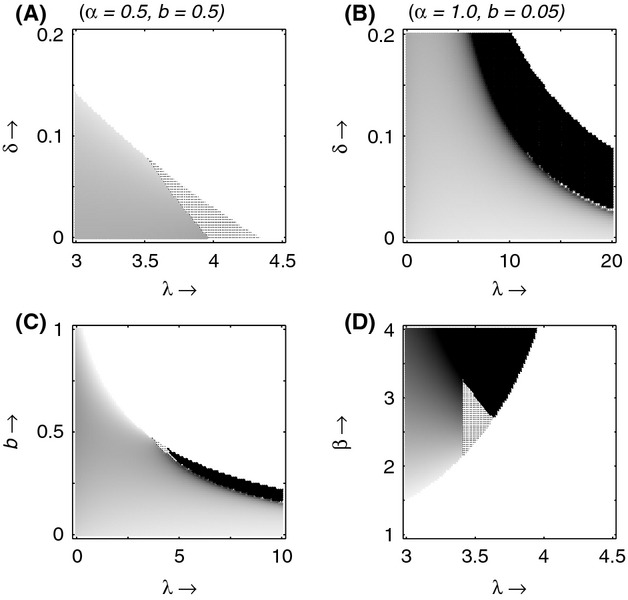
Plot of the evolutionary outcome, with hosts initially having no immunity, *μ* = 0 for varying trade-off curvature, *λ* and epidemiological parameters. Plots (A) and (B) show *λ* versus *δ* (waning immunity), with *α* = 0.5, *b* = 0.5 in (A) and *α* = 1, *b* = 0.05 in (B). Plot (C) shows *λ* versus *b* (death rate) and plot (D) shows *λ* versus *β* (transmission). The shading represents the level of immunity invested in (white = none, black = full) and the dotted region represents branching. Default parameter values are as of Figure 1.

To investigate the impact of the trade-off shape, in particular considering the probability of branching occurring, we again use shaded contour-plots to not only find the location of the singular point in Figure 6, but also to focus on the stability of the point. Note that we shall always assume that the population begins from a point of (*μ* = 0), meaning intermediate repellers will always result in no investment in immunity. First, we plot waning immunity (*δ*) against trade-off curvature (*δ*) in Figure 6A and B. The level of gray shading represents the level of immunity gained (white = none, *μ* = 0; black = full, *μ* = 1) and the dotted section represents evolutionary branching. In the first plot (6A), with intermediate parasite virulence and host lifespan, branching is more likely with low rates of waning immunity, which is expected as if immunity was short-lived then immune hosts would gain little benefit. With high parasite virulence and long-lived hosts (6B), branching again is more likely with low rates of waning immunity, however, this time much stronger decelerating trade-offs are needed for this to occur. In addition, this creates a large region selecting for full immunity (*μ* = 1) for very strong decelerating trade-offs. This is to be expected when the strength of the trade-off curvature is very high, whereby increasing *μ* from low values is very expensive (in terms of lower reproduction), whereas at higher values increasing *μ* further comes at little cost; hence for very strong trade-offs, hosts are more likely to become fully immune or have no immunity than to gain an intermediate level. (In other words, if they can accept the very high initial cost of evolving immunity, then much smaller subsequent costs should be easy to accept.)

We present similar plots for host lifespan (b) against *λ*, and transmission (*β*) against *λ* in plots 6C and D. When considering host lifespan (6C), branching is more likely for stronger trade-offs with hosts possessing an intermediate to long lifespan. When considering transmission (6D) branching is most likely for an intermediate level of transmission, however, only when the trade-off strength is very low; stronger trade-offs result in no investment in immunity. Interestingly, these plots again highlight that full immunity can evolve in the population when lifespan is intermediate to long and transmission is high. In particular very weak, and even linear, trade-offs can lead to full immunity when transmission of the pathogen is high (6D).

## Discussion

An acquired immune memory formed by memory T- and B-cells is common across vertebrate populations and there is widespread interest in how this immune response has evolved (Cooper and Alder 2006; Litman et al. 2010; Hirano et al. 2011). We have studied a mathematical model to consider the ecological and epidemiological conditions that would favor the evolution of costly acquired immunity in host populations. Interestingly, our model has suggested that evolving a fully functioning, highly effective immune response is only likely in specific circumstances. A key requirement for high levels of immunity to evolve is that the costs to investing must be initially rather steep. There is experimental evidence that immune responses cause costs elsewhere in hosts' life-histories (e.g., Moret and Schmid-Hempel 2000), and although clarifying the precise shape of these cost structures is difficult, we would intuitively expect that any initial investment in the required genetic and molecular mechanisms would be particularly costly. Furthermore, hosts must have intermediate or long lifespans for high levels of immunity to evolve. Host lifespans will clearly vary significantly across different species, but we would certainly expect vertebrates to be relatively long-lived compared to invertebrates and plants, where the same form of acquired immunity has not evolved.

It has previously been considered as something of a paradigm that investment in any form of defense should be greatest for long-lived hosts (e.g., Medzhitov and Janeway 1997; Rinkevich 1999). However, in previous theoretical work Miller et al. (2007) found that this was not always the case: if hosts evolved the length of the immune period this result was indeed found to hold, but if hosts could evolve permanent acquired immunity they found that investment was instead highest at intermediate lifespans. Here, we have confirmed and extended the results of Miller et al. (2007), showing that whenever immunity is long lasting, not just permanent, investment in immune memory is greatest at intermediate host lifespans. This is because long-lived hosts need only invest a small amount in immunity to ensure that they are immune for a significant proportion of their lives, but hosts with intermediate lifespans must ensure immunity is achieved early on. However, once immunity wanes at a reasonable rate, this effect weakens and long-lived hosts invest more in immune memory. We therefore highlight that it is the length of the immune period that controls how investment in immune memory varies with lifespan.

More widely, we have found a number of interesting patterns for increased selection for immunity with epidemiological parameters. As may be expected, we have found that high infectivity and disease-induced sterility increase selection for immunity. Perhaps less intuitively, though, we have found that intermediate virulence (disease-induced mortality) selects for higher immunity, as the relative contribution of immune hosts to fitness can be kept higher. This result mirrors previous work on the evolution of clearance (van Baalen 1998) and immune period (Boots and Bowers 2004), suggesting that this is a consistent result for the evolution of immunity. For similar reasons, investment in immunity may also be highest where that immunity is not permanent; a similarly unintuitive result. Where these conditions are not met, we generally predict intermediate levels of defense, and often we find that no immunity at all is selected for. It therefore seems that hosts are only predicted to evolve acquired immunity when faced with pathogens or parasites with specific epidemiological features.

A further way in which full immunity can evolve, in at least part of the population, is through evolutionary branching. In this case, the population initially evolves to an intermediate level of immunity, but then branches in to two distinct sub-populations, one of which then evolves to no immunity (but high reproduction) and the other to full immunity (but low reproduction). Which strain is more prevalent thereafter was found to depend on the epidemiological and ecological conditions. However, we have found that branching occurs for only a small range of trade-off shapes with intermediate initial costs. All vertebrates tested have been found to possess the basic foundations of an adaptive immune system (Cooper and Alder 2006), suggesting that broad branching events have either not occurred or that the nonimmune strain has died out. However, we may speculate that such potential for coexistence in levels of immune memory may have implications for our understanding of the differing adaptive immune systems of jawed and jawless vertebrates, as well as invertebrates and plants.

Our focus here has been on understanding how the epidemiological and ecological environment of hosts affects selection for adaptive immunity. As such we have used a relatively simple one host-one pathogen model to allow for detailed investigation of the dynamics. Of course, as well as immune memory, a key feature of the adaptive immune system is specificity: its ability to recognize not just one but a wide range of antigenic configurations. This specificity is likely to have important consequences for the evolution of adaptive immunity. In particular, if the development of an adaptive immune system gives an increased ability to prevent future infections from multiple pathogens, we may expect it to be much more likely to evolve. However, epidemiological models incorporating multiple pathogen strains have shown the potential for remarkably complex dynamics (Castillo-Chavez et al. 1989; Gupta et al. 1994; Andreasen et al. 1997), and a full evolutionary analysis should be carried out to confirm this intuition. The pathogen itself will also have a considerable role to play in the evolution of immunity. For example, it has been shown that a shorter duration of immune memory can benefit the host by regulating competition between pathogen strains (Wodarz 2003), and that pathogen diversity may impact the diversity of memory cells reserved by the host (Graw et al. 2010). In addition, pathogen coevolution will play a key role in the levels of immunity selected for. These interactions between host and pathogen(s) will be crucial in how immunity has evolved and further theoretical analysis of these questions is necessary to gain greater insights.

Vertebrate hosts exist in a somewhat more complex environment than the standard SIR framework assumes, and factors such as competing species, age-structure and spatial-structure may well have considerable impacts on our results. For example, Bansal and Meyers (2012) considered the impact immunity has in an SIR model on a network, suggesting that higher pathogen virulence may evolve in the spatial model when hosts acquire immunity as they must be more infectious to infect populations multiple times. There are also an array of genetic and molecular components to the development of an adaptive immune system, all of which we have combined together in to one term for immunity. Within-host processes will play a crucial role not just in fighting infection but also in the selection pressures for the development of an immune memory, and combining such processes with our evolutionary ecology approach may reveal further important insights.

Recent years have seen considerable progress in our immunological understanding of the adaptive immune system in vertebrate populations. Here, we have attempted to highlight the importance of ecological and epidemiological processes in shaping the selection pressures for acquired immune memory. Future work should look to incorporate more of the known genetic and molecular features of the adaptive immune system, in particular specificity, in to this evolutionary ecology framework to gain an integrated picture of the evolution of the adaptive immune system.
